# PD52 Evidence Review Of Universal Ultrasound Screening For Developmental Dysplasia Of The Hip In Infants

**DOI:** 10.1017/S0266462324003118

**Published:** 2025-01-07

**Authors:** Arielle Maher, Karen Jordan, Eanan Finnegan, Susan Spillane, Michelle O’ Neill, Máirín Ryan

## Abstract

**Introduction:**

Developmental dysplasia of the hip (DDH) is a congenital disease in which there is abnormal development of the hip in infancy. Ultrasound screening has the potential to enable earlier identification and diagnosis of DDH, facilitating earlier and less invasive treatment. Ultrasound screening programs can be selective or universal, but the optimal method is unclear.

**Methods:**

The aim of this review was to examine the comparative effectiveness of universal and selective ultrasound screening for DDH in infants. The domains of the Health Technology Assessment Core Model® selected for assessment were consistent with a rapid relative effectiveness assessment approach (i.e., focusing on the clinical benefit of the intervention) and included the following: (i) the health problem; (ii) a description of the technology; and (iii) clinical effectiveness and safety outcomes. An expert advisory group comprising nominated representatives from key stakeholder groups was convened for the purposes of quality assurance and to assist in interpreting the evidence.

**Results:**

DDH severity can range from mild dysplasia to complete dislocation, with incidence varying internationally. Ultrasound screening can result in unnecessary treatment given the potential for spontaneous correction of hip instability. Furthermore, treatment may give rise to complications. Appropriate governance of a screening program and associated training may reduce the risk of unnecessary treatment. Limited high quality evidence from four studies was identified. This evidence suggested that increased rates of non-surgical intervention were associated with universal ultrasound screening, compared with selective screening, without a corresponding reduction in the incidence of late DDH or requirement for surgical intervention.

**Conclusions:**

The relative benefit of universal ultrasound screening, compared with selective screening, remains unclear. Screening all infants has the potential to lead to unnecessary treatment, with the risk of clinically significant consequences. Consideration could be given to implementing a selective ultrasound screening program, with appropriate governance, end-to-end care, quality assurance, and outcome monitoring.

